# Dentin dysplasia type I—novel findings in deciduous and permanent teeth

**DOI:** 10.1186/s12903-015-0149-9

**Published:** 2015-12-22

**Authors:** Xin Ye, Kunyang Li, Ling Liu, Fangfang Yu, Fu Xiong, Yun Fan, Xiangmin Xu, Chunran Zuo, Dong Chen

**Affiliations:** Department of Periodontics, School of Stomatology, Zhengzhou University, Zhengzhou, Henan China; Department of Stomatology, The Second Affiliated Hospital of Henan Traditional Chinese Medicine College, Zhengzhou, Henan China; Department of Medical Genetics, Southern Medical University, Guangzhou, China

## Abstract

**Background:**

Dentin dysplasia type I (DD-I) is a rare autosomal dominant hereditary disorder which seriously affects the root development of teeth, causing spontaneous tooth loss (in teenagers). At present, the study of DD-I focuses on familial and phenotypic analyses and reports regarding the ultrastructural study of DD-I are few. The purpose of this study was to clarify and discuss the clinical, histopathological, and ultrastructural features of the dentin defects in DD-I. In addition, the study further explores the root development and provides clues for uncovering virulent genes associated with the disease.

**Methods:**

We recruited 31 members of a four-generation Chinese family, including eleven with dentin defects. Four permanent teeth and four deciduous teeth were obtained from individuals affected by DD-I. At the same time, two caries-free like-numbered permanent teeth and deciduous teeth served as controls, respectively. Analyses of these teeth were carried out using stereomicroscopy, light microscopy, and scanning and transmission electron microscopy, respectively.

**Results:**

Similar to previous reports, extracted teeth showed typical histopathological and ultrastructural features of DD-I and teeth had short roots with obliterated pulp chambers. Furthermore, several novel discoveries were found in teeth affected by DD-I, including; (1) thinner dentin; (2) larger scalloped dentinoenamel junctions; (3) teardrop-shaped lacunae in the enamel; (4) rodless enamel and (5) irregular collagen fibers.

**Conclusions:**

The results exhibited defined features of DD-I in the family and further confirmed that abnormal dentin structure affected both the deciduous and permanent dentitions. In addition, these findings may contribute to a better understanding of the pathogenesis of DD-I as well as aid in the subclassification of this disease.

## Background

Hereditary dentin defects are divided into two main categories, namely dentinogenesis imperfecta and dentin dysplasia (DD) [1, 2]. DD is a hereditary disorder in the formation of dentin that comprises a group of autosomal dominant genetic [3, 4] conditions that are characterized by abnormal dentin structure affecting both the deciduous and permanent dentitions [2–6]. The condition was first described by Ballschmiede [7] but it was Rushton [8] who termed the condition “dentinal dysplasia”. Two types of DD have been identified, and are based on the clinical and radiographic appearance of the affected dentin tissue. DD-I is referred to as “radicular dentin dysplasia” and DD-II as “coronal dentin dysplasia”, in order to indicate the parts of the teeth that are primarily affected by each disorder [9]. A third type of DD or focal odontoblastic dysplasia, with radiographic aspects of the other two types of dysplasia, has also been described [10].

DD-I is a rare anomaly of unknown etiology that affects approximately one patient in every 100,000 [11]. It presents with nearly normal appearing crowns of regular or slightly amber translucency, abnormal spaces between the teeth, malposition and severe tooth mobility. In addition, teeth can be lost prematurely through spontaneous exfoliation related to a lack of root formation [12, 13]. Radiographic examination is a paramount diagnostic tool for identifying this disease, as teeth are characterized by short, blunt and malformed roots, similar to those observed in taurodontic teeth. Other characteristics of the disease include obliteration of all pulp chambers with crescent-shaped pulp remnants parallel to the cementoenamel junction and several periapical radiolucencies in noncarious teeth [3, 4, 6, 14]. Microscopic examination of affected teeth reveals normal enamel with a thin layer of normal dentin adjacent to the dentinoenamel junction and, more centrally, dysplastic dentin masses, suggestive of multiple pulp calcifications [12, 13]. The treatment of patients with DD-I is difficult and strategies such as preventing pulp and periapical infections, and early exfoliation of the teeth have been proposed. Other strategies include dietary analysis and advice, careful oral hygiene, fluoride supplements [15, 16], conventional endodontic therapy retrograde root filling, and periapical curettage [17, 18].

At present, the majority of studies focus on the familial and clinical phenotypic analyses of DD-I, and there are few reports regarding the ultrastructural study of DD-I. This is due to the low incidence of the disease and thus the difficulty in obtaining teeth affected by DD-I *in vitro*. The purpose of this study was to examine the histology and ultrastructure of teeth affected by DD-I and to evaluate developmental models which can explain the findings.

## Methods

The study protocol and patient consents were reviewed and approved by the Institution Review Boards at Zhengzhou University. We recruited 31 members of a four-generation Chinese family, including eleven with dentin defects. All available family members were examined by clinical examination and panoramic radiograph to confirm the diagnosis of DD-I. Four permanent teeth and four deciduous teeth were collected from people of the family affected by DD-I. At the same time, two caries-free like-numbered permanent teeth and two healthy like-numbered deciduous teeth served as controls. All the subjects had no history of serious systemic disease. Teeth were initially fixed in 10 % formalin and then stored in 70 % ethanol. Specimens were photographed, and longitudinal, undecalcified, buccolingual sections were cut with a slow-speed diamond saw (Leitz,Stuttgart,Germany) microtome to 1 mm.

Stereomicroscopy: Sections were polished with abrasive paper (7000) and then observed with stereomicroscope.

Light microscopy (LM): Sections were washed in distilled water, and were polished with abrasive paper (800; 1000; 2000; 3000 and 7000) at a depth of 15 μm. The surfaces obtained were etched with 40 % phosphoric acid for 30 s and gradually dehydrated before staining with neutral balsam for standard LM analysis.

Scanning electron microscopy (SEM): Sections were polished with abrasive paper (1000; 3000 and 7000). The surfaces were then etched with 40 % phosphoric acid for 40s, and progressively dehydrated before coating with a gold–palladium alloy for SEM examination using a Quonxe-2000(PHILIPS,Amsterdam,Netherlands)at 25 kV. SEM stereoscan with magnifications of 200×, 500×, 1000×, 2000×, and 5000× were used, respectively.

Transmission electron microscopy (TEM): Fractured portions of several samples were decalcified using 0.1 M ethylenediaminetetraacetic acid disodium salt, pH 7.3–7.5, for one and a half months at room temperature and pressure. Ultra-thin sections were made using a diamond knife and examined with a JEM-1400 TEM(JEOL, Tokyo, Japan)operating at 80 kv.

This study was approved by the Ethics Committee of Zhengzhou University. Informed consent for research participation and image publication was obtained for each participant.

## Results

**Fig. 1:**
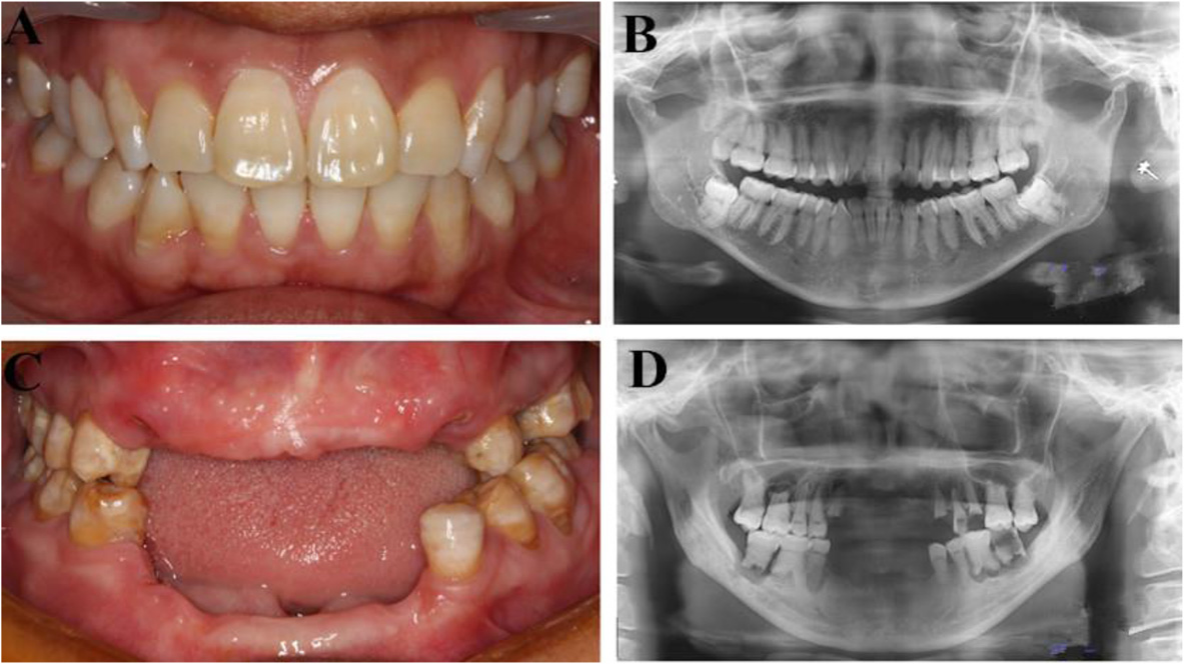
Intraoral images and panoramic radiographs of individuals. **a** Intraoral image of a normal subject. **b** Panoramic radiograph of normal subject. **c** Intraoral photo of a 47 year old subject affected by DD-. **d** Panoramic radiograph of a subject affected by DD-I

**Fig. 2:**
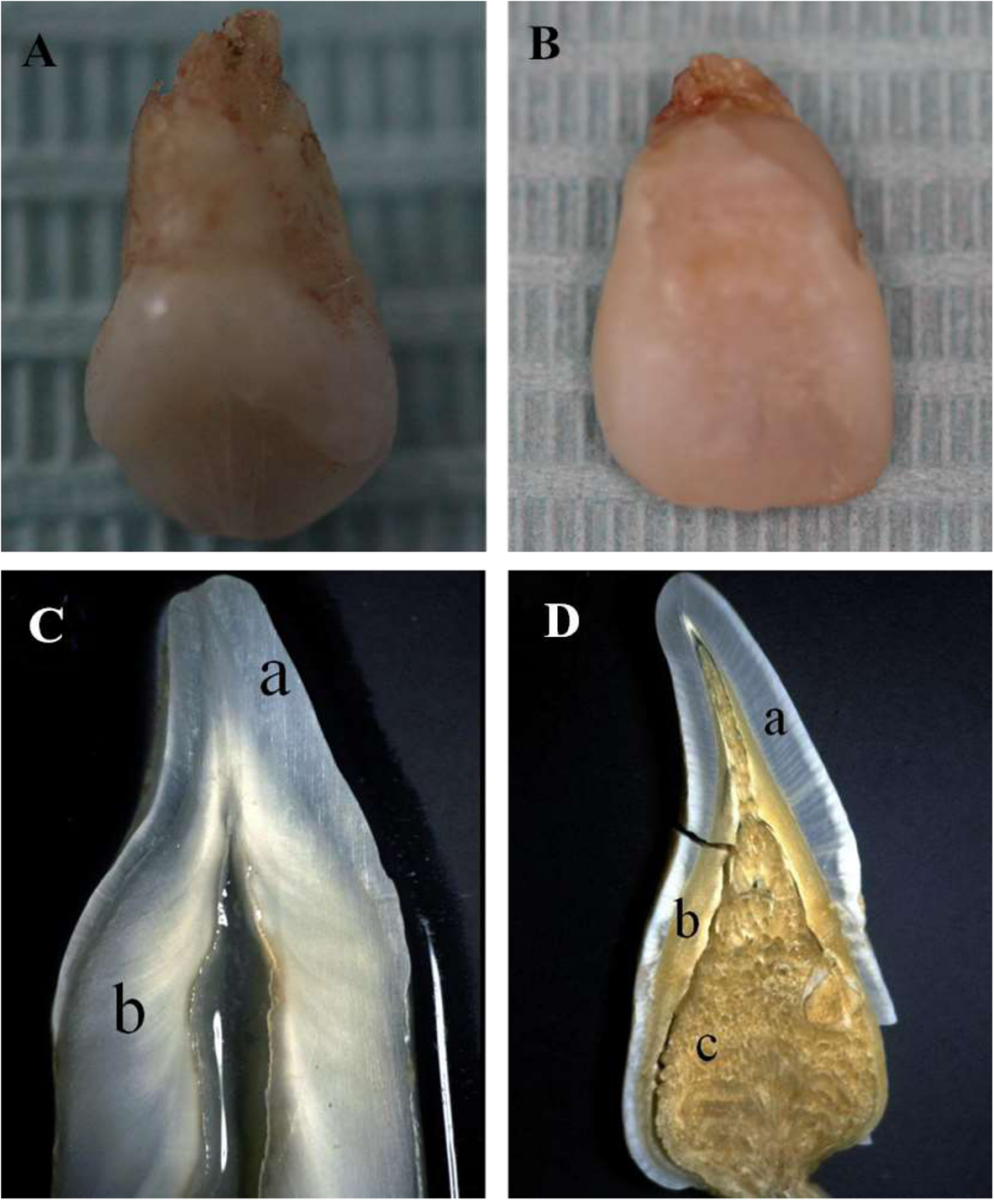
Clinical and stereomicroscopic observations of normal and DD-I affected teeth. **a** A deciduous tooth affected by DD-I and (**b**) a permanent tooth from the subject affected by DD-I. Examples of a (**c**) normal permanent tooth and (**d**) a permanent tooth affected by DD-I

**Fig. 3:**
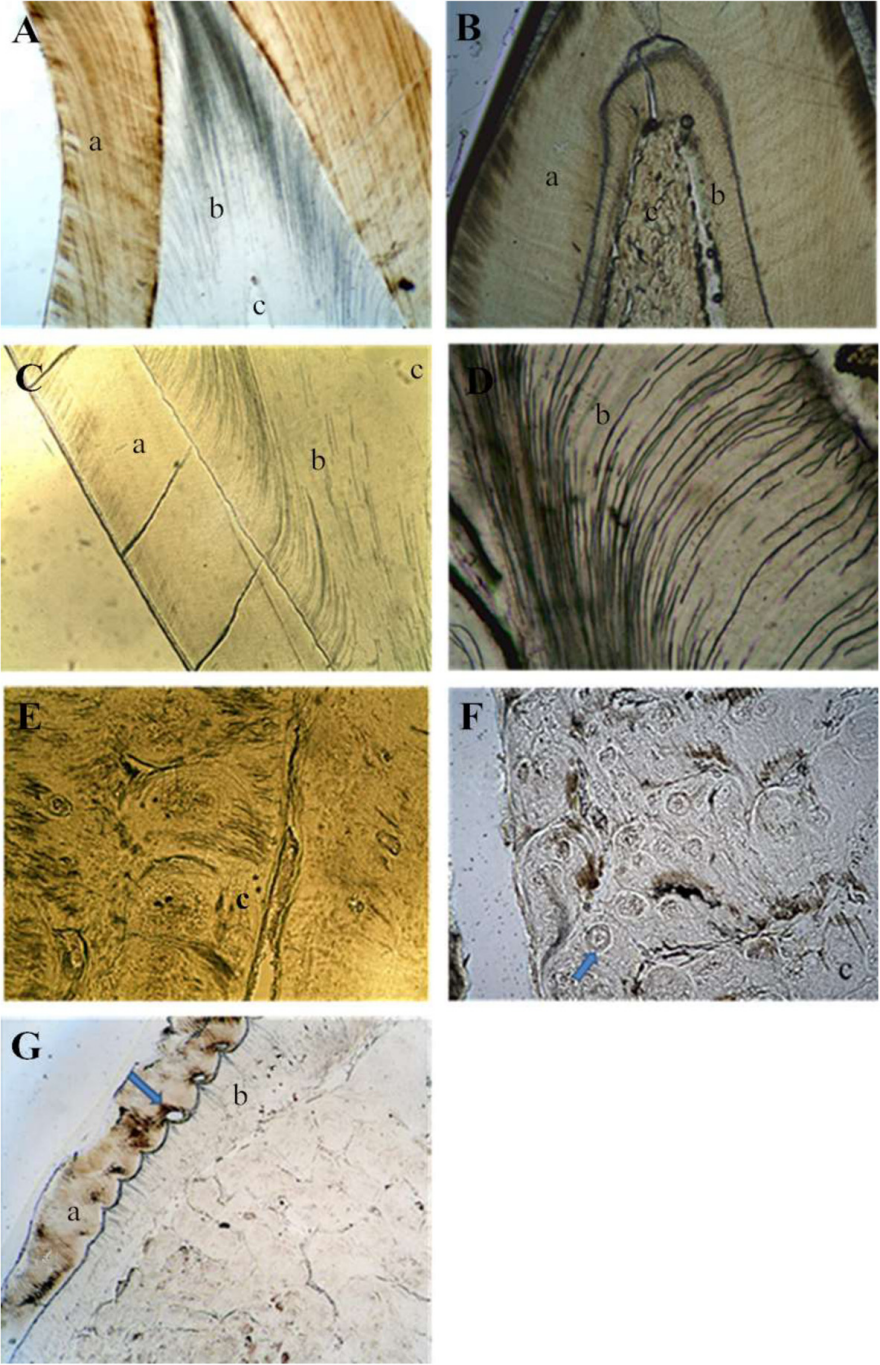
LM examination of the teeth specimens. **a** Ground section of a control permanent tooth (40×). **b** Ground section of a permanent tooth affected by DD-I: normal-appearing enamel, thin dentin and large pulp filled with irregular organization are present (40×). **c** Well-demarcated mantle dentin continued with fractured and discontiguous and sparse dentinal tubules were found in deciduous teeth affected by DD-I (100×). **d** Dentin of a permanent tooth affected by DD-I (400×). **e** “Stream flowing around boulders” patterns were found in deciduous teeth affected by DD-I (200×). **f** A permanent tooth affected by DD-I (the arrow indicates a dentin bead) (400×). **g** Teardrop-shaped lacunae near the cervical enamel were found in permanent teeth affected by DD-I (the arrow indicates a lacuna) (100×)

Clinical examination of the individual affected by DD-I revealed that the maxillary and mandibular incisors were lost, and there was severe mobility of the residual teeth, although teeth retained normal shaped crowns (Fig. 1c). Radiographic examination identified characteristic features associated with DD-I including, residual teeth with short roots, obliteration of pulp chambers with crescent-shaped pulp remnants and periapical cysts without carious lesions (Fig. 1d). Extracted teeth of individuals affected by DD-I exhibited generalized normal shaped crowns but with a slightly yellow color with short roots, obliterated pulp chambers as well as defective enamel (Fig. 2a, b). Examination of ground sections of DD-I and normal teeth under a stereomicroscope and LM revealed that the enamel was of normal thickness in both normal and DD-I teeth and a normal-appearing enamel was continuous with a normal-appearing coronal dentin band of tubules (Figs. 2c, d; 3a, b). In addition, well-demarcated mantle dentin was found in several areas of all DD-I teeth examined (Fig. 3c, d), and much of the dentin directly adjacent to the enamel had dentinal tubules and a distinctly-differentiated mantle dentin. However, dentinal tubules of circumpulpal dentin were fewer compared to the controls, which were fractured and discontiguous in certain areas (Fig. 3c, d), and the thickness of dentin was noticeably thinner in DD-I teeth compared to the controls (Figs. 2d, 3b). Furthermore, and more importantly, the pulp chamber was extra-large and the entire region was fused with large masses of irregular organizations (Figs. 2d, 3b). Analysis at a higher magnification revealed that the dysplastic dentin masses consisted of mineralized whorled dentin globules and dentin tubules with the two sections forming a characteristic “stream flowing around boulders” root dentin structure (Fig. 3e, f). Further observation revealed that the dentinoenamel junction was either smooth or scalloped, but the scalloped structure of DD-I was larger than that of controls (Fig. 3g). In addition, teardrop-shaped lacunae were found near the cervical enamel in DD-I teeth (Fig. 3g).

**Fig. 4:**
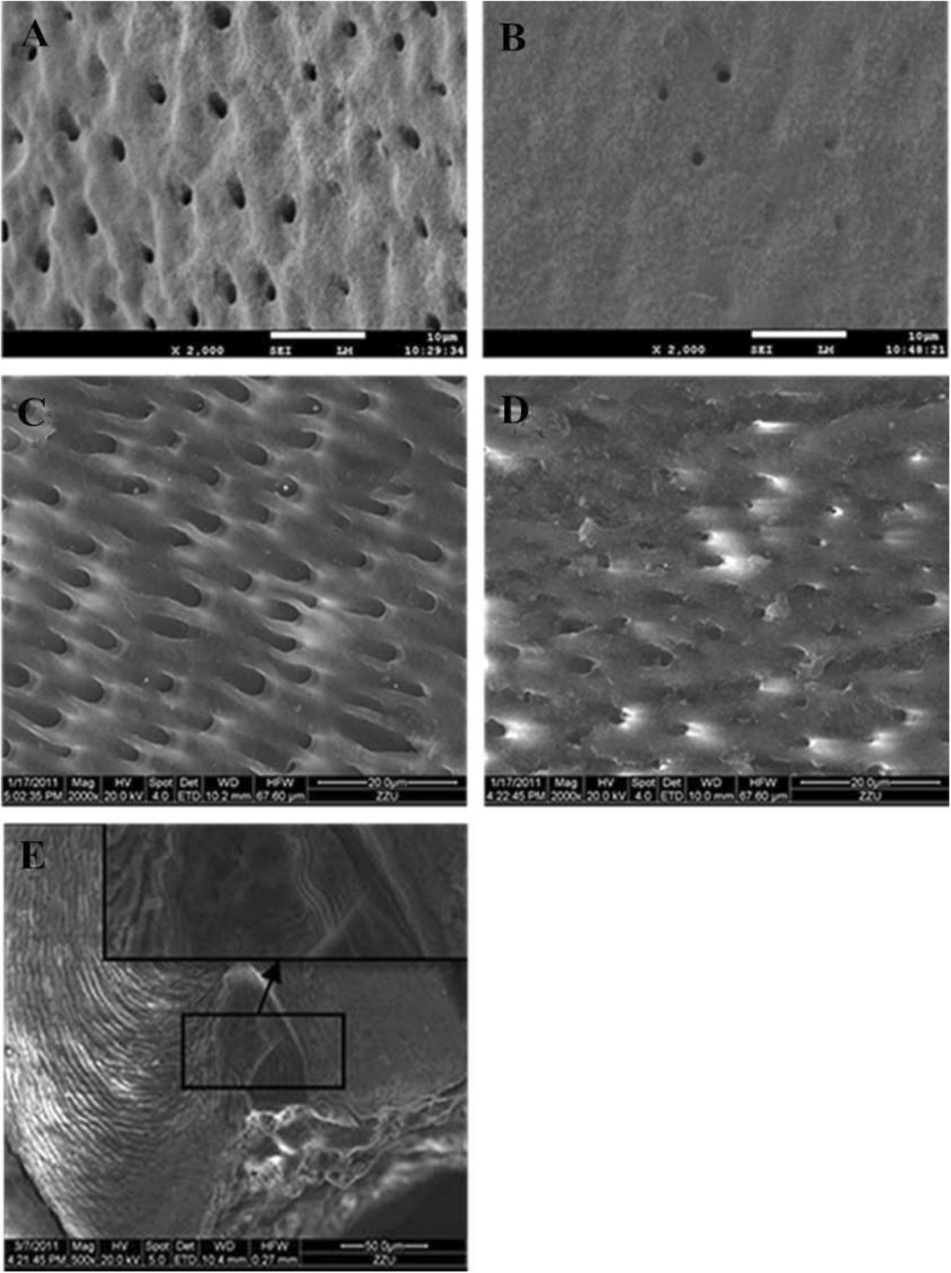
SEM examination of the teeth specimens. **a** Regularly arranged dentin tubules were found in normal deciduous teeth (2000×). **b** Sparsely scattered dentin tubules with reduced diameter were found in deciduous teeth affected by DD-I (2000×). **c** Dentin tubules in a normal permanent and (**d**) a permanent tooth affected by DD-I (2000×). **e** Rodless enamel with black and white horizontal stripes in a permanent tooth affected by DD-I (500×)

**Fig. 5:**
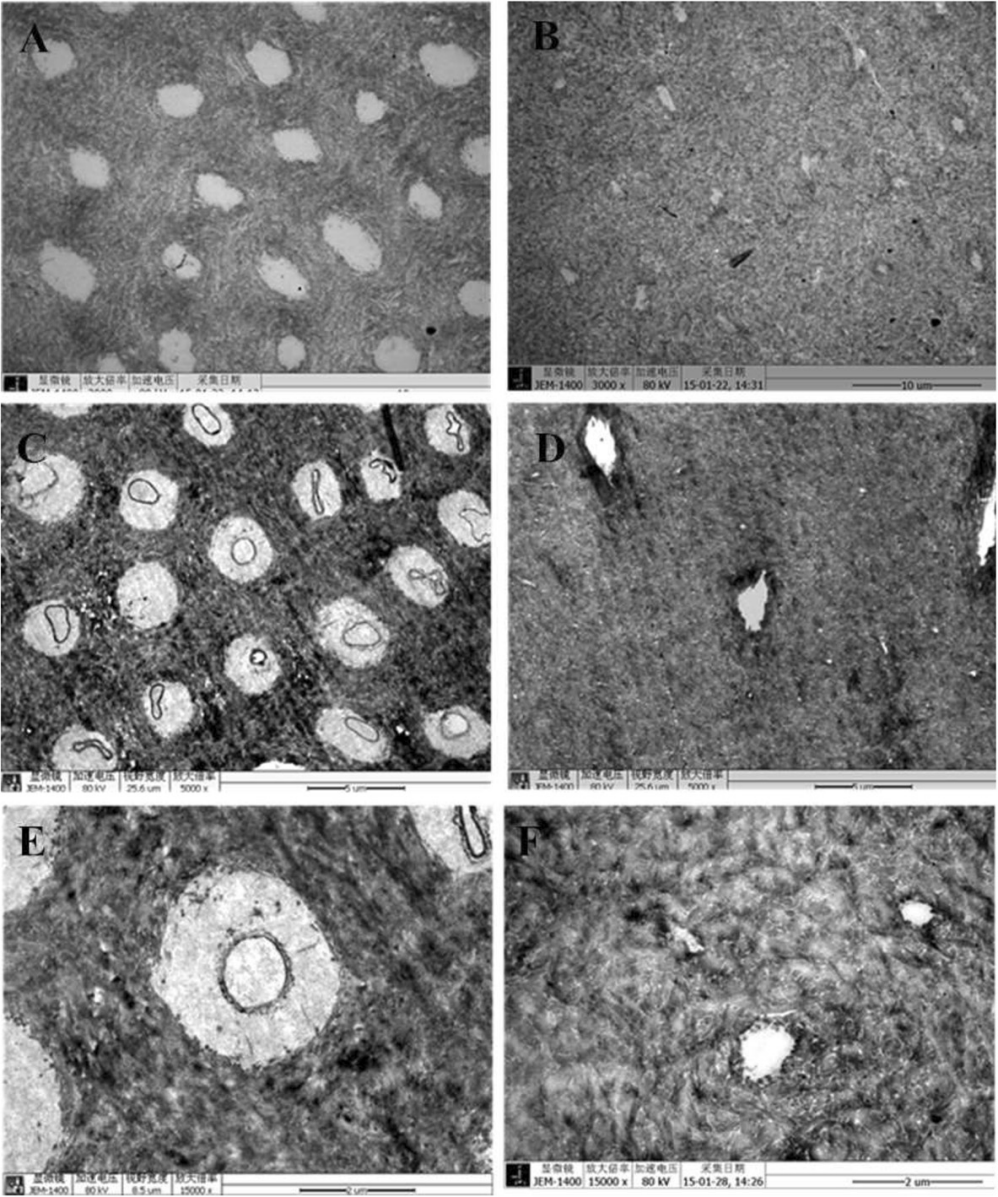
TEM examination of the teeth specimens. **a** Normal inner dentin with orderly dentinal tubules having no sheath-like structures were found in control deciduous teeth (3000×). **b** Tubules with irregular shape, size, and pattern of organization were found in deciduous teeth affected by DD-I (3000×). **c** Outer dentinal tubules with sheath-like structures of normal permanent teeth and (**d**) dentin tubules of permanent teeth affected by DD-I (5000×). **e** Laminar-type collagen in a normal tooth and (**f**) ill-defined margins and irregular collagen fibers in a tooth affected by DD-I (15,000×)

SEM of the enamel revealed that dentinal tubules of the controls were dense and homogeneous (Fig. 4a, c). However, dentinal tubules were fewer in number and smaller in diameter in DD-I teeth than in normal teeth, and some aperture of dentinal tubules of DD-I teeth were occluding (Fig. 4b, d). At higher magnifications, DD-I teeth contained regions of rodless enamel with black and white horizontal stripes (Fig. 4e). TEM revealed that, in control teeth, a collagen-rich dentin matrix was surrounded by well demarcated dentinal tubules which were regularly distributed, equal-sized and which had an approximately circular cross-section. The tubules of control teeth also appeared empty or with a minute quantity of electron dense remnants of intratubular contents (Fig. 5a, c) and, in addition, laminar-type collagen could be found between the intertubular dentin (Fig. 5e). In contrast, DD-I specimens had different amounts of collagen fibers that were located around the tubules with variable configuration, diameter, and intertubular distance. When seen, the dentinal tubules usually had a smaller diameter and a more irregular outline than the tubules found in the controls and the margin of the tubule wall was poorly demarcated. Intratubular content varied from a granular material to a dense amorphous mass that was present in most of the tubules, and with increasing abnormality, the tubules became sparse and small, and finally vanished (Fig. 5b, d). In addition, the collagen fibers appeared short and intruded into the tubules, and areas with sparse collagen became more frequent. Finally, collagen fibers were also haphazardly oriented in the dentin matrix (Fig. 5f). (A summary of the main results can be found in Table. 1)

**Table 1:** Summary of the main results

	Normal permanent teeth	DDI-permanent teeth	Normal deciduous teeth	DDI-deciduous teeth
Normal crowns	+	+	+	+
Rootless teeth		+		+
Periapical radiolucencies		+		+
Pulp obliteration		+		+
Partially obliterated crescent shaped pulp chamber		+		+
Normal enamel	+	+	+	+
Well-demarcated mantle dentin	+	+	+	+
Dysplastic dentinal tubules		+		+
“Stream flowing around boulders” root dentin structure		+		+
Chaotic collagen fibers		+		+
Larger scalloped dentinoenamel junctions		+		
Teardrop-shaped lacunae in the enamel		+		
Rodless enamel		+		

## Discussion

DD-I is an autosomal dominant hereditary disturbance in dentin formation, which may present with either mobile tooth movement or pain associated with spontaneous dental abscesses or cysts [11, 19–22]. In the current study, examination of teeth from individuals with DD-I revealed defined features of the disease, including clinically normal crowns, increased tooth mobility, radiographic obliteration of pulp chambers, periapical radiolucent lesions, and short blunted roots. DD-I is divided into four subtypes (a–d) based on the amount of obliteration of the pulp chambers, root development, and periapical radiolucent areas. DD-Ia is characterized by complete obliteration of the pulp, no root development, and many periapical radiolucent areas. In DD-Ib, a single small horizontally oriented and crescent shaped pulp is present, roots are only a few millimeters in length and there are frequent periapical radiolucencies. DD-Ic is characterized by the presence of two horizontal or vertical crescent shaped pulpal remnants which surround a central island of dentine. In addition, teeth have shortened root lengths and variable periapical radiolucencies. Finally, in DD-Id, the pulp chambers are distinct, with pulp stones in the coronal third of the root canal (with corresponding bulging of the root). Additionally, the roots have significant development, and there are few, if any, periapical radiolucencies [21]. In the present study we also found that most DD-1 affected teeth were free from caries. The reason for this finding could be explained by a number of factors including, good oral hygiene practice of the patient, a high fiber diet, early loss of rootless teeth resulting in a shortening of the adherence time of caries-causing bacteria, or a combination of these actors. Further research is needed to confirm the factors associated with the above finding.

In our study, variations in dentin structures were observed with stereomicroscopic, LM, SEM and TEM analyses and there were six important new phenotypic variations found; (1) The thickness of dentin was thinner compared to the controls; (2) the dentinoenamel junction (DEJ) was either smooth or scalloped, but the scalloped structure found in DD-I teeth was larger than controls; (3) teardrop-shaped lacunae were found near the cervical enamel; (4) regions of rodless enamel with black and white horizontal stripes could be found in DD-I teeth and (5) the collagen fibers were irregular.

Melnick et al. observed that dentinal tubules in teeth of DD-I patients appeared to be fewer in number and generally smaller in diameter than that found in normal teeth [23]. Results from LM, SEM and TEM analyses in our study revealed that dentinal tubules in DD-I specimens were either greatly reduced in number or smaller in diameter than control specimens. Of greater significance, in some pathological areas we could not find dentinal tubules and this could be explained by degradation and dysfunction of odontoblasts [24]. We hypothesize that dentinal tubules are important in dentinogenesis and they may act as pathways of transport for matrix gel protein and subsequent reabsorption of the gel and transport of calcium phosphates during dentin mineralization. The negative effects on the mineralization of peritubular dentin and intertubular dentin found in this study could therefore have resulted from decreased tubules [25]. The results of thinner dentin and the structure of “stream flowing around boulders” may be explained as follows: An initial normal function of the odontoblasts or preodontoblasts can be seen by a well-demarcated mantle dentin layer [26], but if odontoblasts become dysfunctional this results in a quick, chaotic matrix deposition and mineralization, finally leading to pulp obliteration [24]. This new matrix is likely to be secreted by odontoblast-like cells which are derived from undifferentiated pulp mesenchyme cells following the apoptosis or necrosis of the original odontoblasts. These odontoblast-like cells, with an altered genetic program of matrix production, continuously secrete matrix gel protein and calcium phosphate resulting in pulp chamber or root canal obliteration [4, 11, 24, 26]. The DEJ can be regarded as a biological adhesion interface between enamel and dentin. The configuration of the DEJ was scalloping rather than a straight line in DD-I teeth. This connection, increasing the enamel and dentin contact area, was advantageous to the firm combination of the two proteins [27]. According to our observations, the scalloped structure of DD-I teeth was larger than in controls and at the same time, results from LM analyses revealed that teardrop-shaped lacuna near the cervical enamel corresponded to regions of rodless enamel. This suggested that the structures of enamel in DD-I teeth may change, although this change in enamel was not extensively found in the current study. In this regard, more studies are needed to determine whether this alteration was an exceptional manifestation of DD-I teeth resulting from a specific mutation, or an artifact of specimen processing. In support of a possible enamel change, there are previous reports of TEM studies on teeth affected by DD-I in which the collagen fibers displayed extreme variation in their organizational pattern. In regards to improper mineralization of the secreted dentin matrix being a characteristic of dentin disorders, two different mechanisms could be responsible: First, in the collagenous forms of DD-I, a defective nucleation of apatite crystals at the collagen surface may be the result of a reduction in collagen as well as poor structural quality of collagen molecules [28]. Second, in the non-collagenous forms of DD-I, abnormal functioning of two unique dentin matrix proteins, dentin sialoprotein and dentin phosphoprotein, may interfere with normal dentin matrix mineralization [29]. Thus, abnormalities in the non-collagenous matrix components cannot be excluded in the pathogenesis of DD-I.

The exact mechanism responsible for DD-I is an enigma, although numerous theories have been proposed. Logan et al. [12] proposed that the dentinal papilla which is responsible for the abnormalities in root development becomes calcified, resulting in reduced growth and final obliteration of the pulpal space. Sauk et al. [13] suggested a problem in the epithelial component of the developing tooth germ, in which the invagination of the root sheath occurs too soon, and in a sequence of futile attempts to correct itself, results in abnormal dentin formation. Witkop [9] proposed that the dysplasia results from epithelial cells from the sheath of Hertwig breaking off and migrating into the dental papilla, where they produce ectopic dentin formation. Wesley et al. [30] offers as an alternative hypothesis, that an error in the continual induction of odontoblasts after the interaction with the ameloblastic layer causes differentiation and/or abnormal function of the odontoblasts. On the basis of our results, we support the idea of Wesley and colleagues. Further histopathological and ultrastructural studies of dentin in DD-I teeth may help to obtain a better understanding of the pathogenesis, as well as the clinical phenotypes of DD-I.

## Conclusions

We have studied the clinical, histological and ultrastructural structures of teeth in a family with dentin defects and all results indicate defined features of DD-I. Novel findings from this study include (1) thinner dentin; (2) larger scalloped dentinoenamel junctions; (3) teardrop-shaped lacunae in the enamel; (4) rodless enamel and (5) irregular collagen fibers, all of which contribute to the pathology of the disease. Furthermore, these findings may contribute to a better understanding the pathogenesis, as well as aid in the subclassification of DD-I.

## Abbreviations

DGI: Dentinogenesis imperfecta
DD: Dentin dysplasia
LM: Light microscopy
SEM: Scanning electron microscopy
TEM: Transmission electron microscopy
EDTA-2Na: Ethylenediaminetetraacetic acid disodium salt
DSP: Dentin sialoprotein
DPP: Dentin phosphoprotein
